# Optically and acoustically triggerable sub-micron phase-change contrast agents for enhanced photoacoustic and ultrasound imaging

**DOI:** 10.1016/j.pacs.2017.04.001

**Published:** 2017-04-11

**Authors:** Shengtao Lin, Anant Shah, Javier Hernández-Gil, Antonio Stanziola, Bethany I. Harriss, Terry O. Matsunaga, Nicholas Long, Jeffrey Bamber, Meng-Xing Tang

**Affiliations:** a Department of Bioengineering, Imperial College London, London, UK; b Joint Department of Physics and CRUK Cancer Imaging Centre, The Institute of Cancer Research and The Royal Marsden NHS Foundation Trust, London, England, UK; c Department of Chemistry, Imperial College London, London, UK; d Department of Medical Imaging, University of Arizona, Tucson, AZ, USA

**Keywords:** Phase-change contrast agent, Droplet, Microbubble, Ultrasound, Photoacoustic, Optical/acoustic vaporisation, Multispectral optoacoustic tomography (MSOT)

## Abstract

We demonstrate a versatile phase-change sub-micron contrast agent providing three modes of contrast enhancement: 1) photoacoustic imaging contrast, 2) ultrasound contrast with optical activation, and 3) ultrasound contrast with acoustic activation. This agent, which we name ‘Cy-droplet’, has the following novel features. It comprises a highly volatile perfluorocarbon for easy versatile activation, and a near-infrared optically absorbing dye chosen to absorb light at a wavelength with good tissue penetration. It is manufactured via a ‘microbubble condensation’ method. The phase-transition of Cy-droplets can be optically triggered by pulsed-laser illumination, inducing photoacoustic signal and forming stable gas bubbles that are visible with echo-ultrasound *in situ*. Alternatively, Cy-droplets can be converted to microbubble contrast agents upon acoustic activation with clinical ultrasound. Potentially all modes offer extravascular contrast enhancement because of the sub-micron initial size. Such versatility of acoustic and optical ‘triggerability’ can potentially improve multi-modality imaging, molecularly targeted imaging and controlled drug release.

## 1 Introduction

Microbubble ultrasound contrast agents have been widely used as a valueable imaging tool in clinical radiology and cardiology [1]. At the same time there continues to be extensive research efforts focusing on new paradigms for contrast-enhanced ultrasound imaging (CEUS) [2], [3], and microbubble-mediated therapy [4], [5]. However, these micron-sized microbubbles are limited to the intravascular space [6]. As a means of exploring the extravascular space, sub-micron phase-change droplets show widespread interest [7], [8]. They can potentially extravasate the ‘leaky’ cancerous vasculature into interstitium [9] prior to vaporisation, providing extravascular contrast enhancement upon the phase transition of droplets to echogenic microbubbles. Vaporisation can be triggered either acoustically or, for droplets containing an optical absorber, optically. The optical activation of phase-change droplets can provide photoacoustic contrast enhancement [10].

Existing studies on such dual-modality contrast agents have demonstrated the generation of both optical and ultrasound contrast after optical activation [10], [11], [12], [13], [14], [15], [16]. However these studies did not explore the option of acoustic activation. This would add versatility of vaporisation triggering, offering new possibilities in dual mode imaging, molecular imaging and drug delivery. Furthermore, high boiling point (b.p.) perfluorocarbons were used in these studies, i.e., perfluoropentane (b.p. 29 °C) [10], [11], [12], [17], [18] and perfluorohexane (b.p. 56 °C) [13], [14], [19]. A low b.p. may be preferred, to minimise un-wanted bioeffects [20], especially when activating in deeper tissues. Although Dove et al. [21] engineered optically triggered droplets using a low b.p. perfluorocarbon (decafluorobutane, DFB, b.p. −2 °C), the optical absorber employed (i.e. gold spheres) may limit the imaging depth due to the weakly penetrating plasmonic resonance wavelength (i.e. 535 nm).

In this study, we have employed an easily vaporisable sub-micron, phase-shift droplet made with a highly volatile perfluorocarbon and formulated via condensation of pre-formed, lipid-shelled microbubbles. This has previously shown great promise as an extravascular contrast agent for diagnostic and therapeutic ultrasound [6], [8], [22], [23], [24], [25], [26], including promise for eventual clinical translation [27], [28]. In this paper, we provide the first demonstration of its potential for photoacoustic imaging, and thus as a versatile three-mode agent. We developed and characterised a new sub-micron phase-change droplet (Cy-droplets) by incorporating a near-infrared (NIR) optical absorber, i.e., a Cyanine7.5 bioconjugate, into the precursor microbubble membranes before condensation. Cyanine7.5 has a peak absorption at a wavelength of 788 nm, offering relatively good tissue penetration [29]. Here we demonstrate that the Cy-droplet phase transition can either be triggered by a pulsed laser to produce substantial photoacoustic signal enhancement as well as subsequent ultrasound contrast, or be triggered acoustically using clinical ultrasound pulses to provide conventional ultrasound contrast.

## 2 Methods

### 2.1 Cyanine7.5 bioconjugation synthesis

**Fig. 1:**
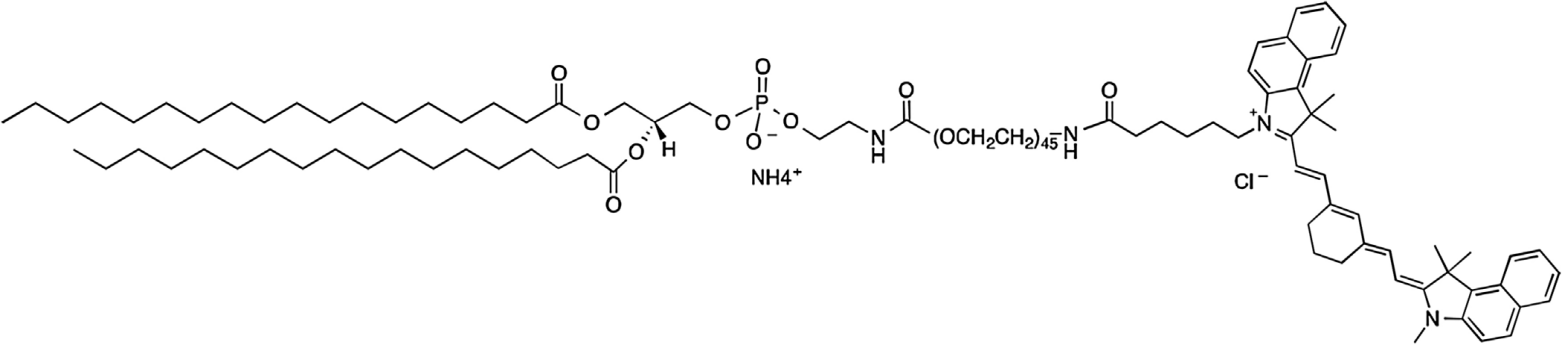
Structure of the functionalised DSPE–PEG (2000)–Cyanine7.5 phospholipid.

Cyanine7.5 NHS ester (Lumiprobe GmbH, Germany) was conjugated to the amine terminus of a commercially available phospholipid with a PEG2000 spacer (DSPE-PEG(2000)-NH_2_) via a NHS-mediated coupling reaction to afford the target Cyanine7.5 dye-functionalised phospholipid (DSPE–PEG(2000)–Cyanine7.5) after purification by dialysis. DSPE–PEG(2000)–Cyanine7.5 (Fig. 1) was characterised by various analytical and spectroscopic techniques (see Supporting information, Figs. S1–S2). In a typical reaction, DSPE–PEG(2000)–NH_2_ (3.0 mg, 1.1 μmol) and 6 μL of triethylamine were dissolved in 120 μL of dry DMSO. To this solution, 120 μL of DMSO containing Cyanine7.5 NHS ester (1.7 mg, 2.2 μmol) was added dropwise. The resulting mixture was allowed to react at room temperature overnight under continuous stirring. Distilled water was then added to the reaction mixture. The solution was centrifuged, and the supernatant was passed through a 0.45 μm filter to remove insoluble traces. The supernatant was then dialysed using a Spectra-Por^®^ Float-A-Lyzer^®^ G2 (Sigma-Aldrich, Milwaukee, Wis) (MW cutoff of 3.5–5 kDa) against water (3 × 500 mL). The dialysate, containing the pure product, was lyophilised and the residue dried in vacuo over P_2_O_5_. All lipids used in this study were purchased from Avanti Polar Lipids, Inc., USA.

### 2.2 Cy-droplets synthesis

**Fig. 2:**
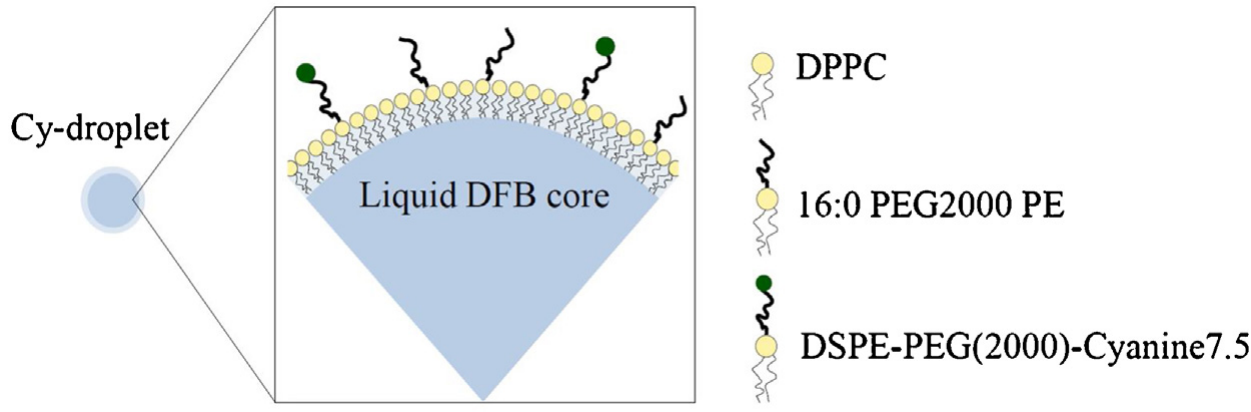
Schematic showing the composition of Cy-droplet contrast agent.

The lipid-coated, DFB-filled precursor Cy-microbubbles were manufactured using a modified formulation described by Sheeran et al. [6]. Briefly, the lipid mixture consisted of 1,2-dipalmitoyl-sn-*glycero*-3-phosphocholine (DPPC), 1,2-dipalmitoyl-sn-*glycero*-3-phosphoethanolamine-N-[methoxy(polyethylene glycol)-2000] (16:0 PEG2000 PE) and DSPE-PEG(2000)-Cyanine7.5 9:0.8:0.2 (Fig. 2), m:m:m (total lipid concentration of 0.85 mg/mL) dissolved in a solution of propylene glycol, glycerol, and phosphate-buffered saline (PBS) (15/5/8, v/v/v). The Cyanine7.5 concentration in the lipid solution was 13 nM. Next, 1 mL of the resulting lipid solution was sealed in a 2 mL glass vial and the headspace was then purged with DFB at room temperature. The amount of DFB used to synthesise the Cy-droplets (Fig. 2) was approximately 8.8 × 10^−10^ mL per particle. The precursor Cy-microbubbles were produced via mechanical agitation. Finally, the Cy-droplet emulsion was obtained by condensing Cy-microbubbles using the method of Li et al. [22].

### 2.3 Controls

Control samples included six groups: (1) precursor Cy-microbubbles, (2) blank-microbubbles, (3) blank-droplets, (4) Cy-solution, (5) blank-droplets in Cy-solution, (6) deionised water (used to dilute all the experiment samples). The lipid-shell compositions of both blank-microbubbles (precursor) and blank-droplets were prepared in an identical fashion except for the lipid composition, which consisted of DPPC, 16:0 PEG2000 PE in a molar ratio of 9:1. The Cy-solution was prepared using a similar procedure to the precursor Cy-microbubble lipid solution, by substituting the lipid mixture with Cyanine7.5 NHS ester powder and dispersing in the aforementioned propylene glycol, glycerol, and phosphate-buffered saline diluent mixture. The concentration of Cyanine7.5 dye was kept the same across all the controls and Cy-droplet emulsion.

### 2.4 Characterisation of precursor Cy-microbubbles and Cy-droplets

The precursor Cy-microbubbles and Cy-droplets were observed using both bright-field optical and confocal microscopy. Confocal microscopy (Leica SP5 DMI 6000 CS, 60 × objective) was operated to locate the fluorescence from precursor Cy-microbubbles and Cy-droplets. Due to resolution limitations of those microscopes, only size outliers of Cy-microbubbles and Cy-droplets could be visualised to determine optical appearance and the location of fluorescence. One hundred μL aliquots of diluted (1:100) stock Cy-microbubbles and Cy-droplet samples were imaged at a plane through the cross section of the samples. The imaging slice thickness was set to 0.76 μm. For bright-field microscopy (Nikon Eclipse 50i, 40 × objective), 10 μL diluted samples were first introduced into a haemocytometer and then sized and counted according to the protocol detailed in Sennoga et al. [30]. The size distribution of Cy-droplets was measured using dynamic light scattering (DLS, Malvern Nano ZetaSizer, UK). Before measurement, the DLS was calibrated using Latex particles with a mean diameter of 750 nm. Following calibration, 10 μL of the stock Cy-droplet emulsion sample was diluted in 90 μL milliQ water (milliQ, Canada) in order to measure the droplet size. The absorption spectra of the Cy-droplet lipid solutions were then measured using a UV/VIS spectrometer (Lambda 25, PerkinElmer, UK).

### 2.5 Preparation of tissue mimicking phantom for *in-vitro* experiments

**Fig. 3:**
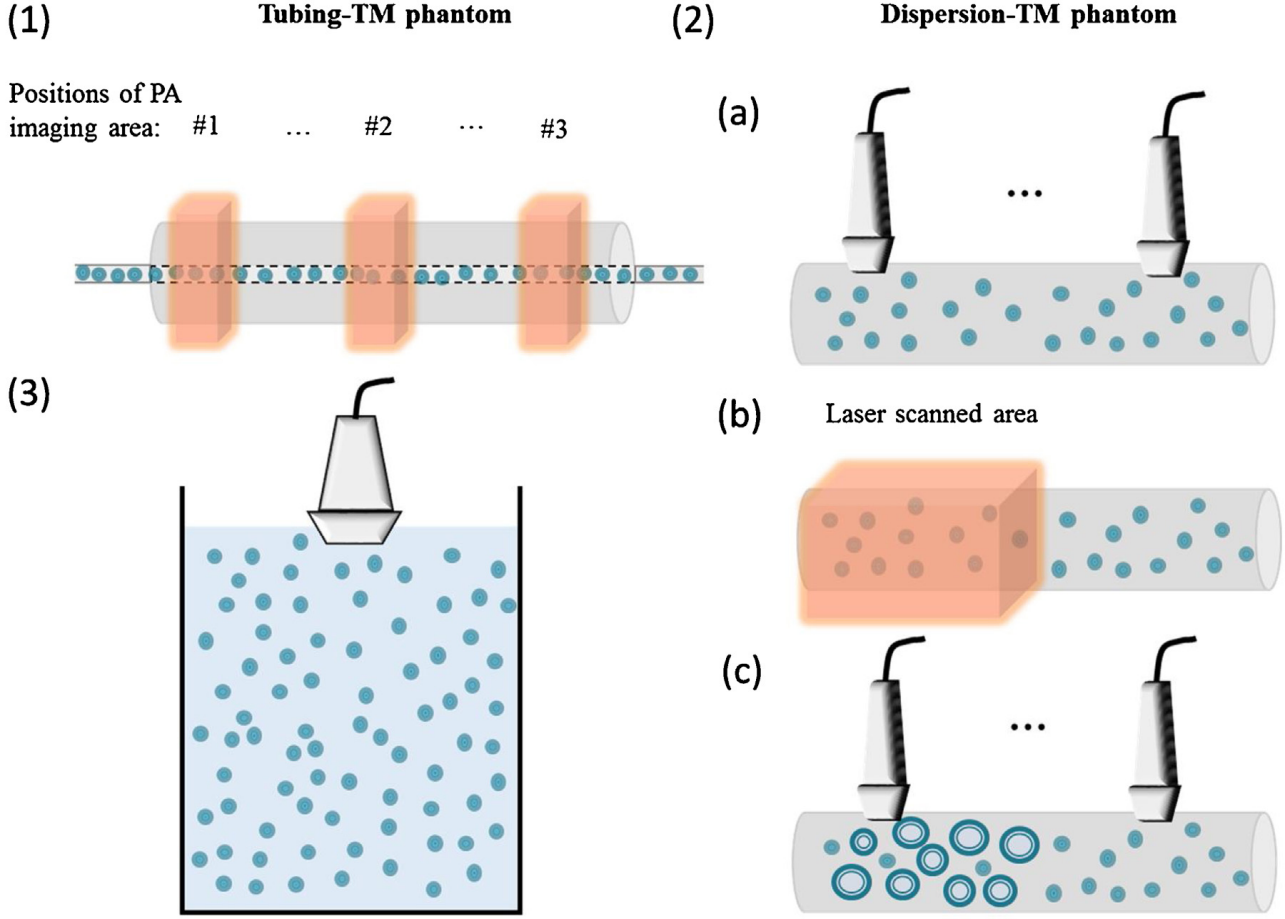
(1) Schematic of the tubing-TM phantom used for photoacoustic signal acquisition (not to scale). (2) Ultrasound imaging and MSOT laser illumination with the dispersion-TM phantom. (3) Experimental setup for acoustic activation of Cy-droplets and blank-droplets.

Two types of tissue mimicking (TM) agar phantoms were prepared for optical vaporisation experiments. A Tubing-TM phantom (Fig. 3(1)) was used for photoacoustic signal acquisition by embedding a semi-transparent silicone tube (ID = 1.5 mm OD = 1.9 mm, Harvard Apparatus, UK) in the centre of the cylindrical agar-intralipid TM phantom (diameter = 15 mm, length = 10 cm). The agar-intralipid gel was manufactured following the protocol adapted from Madsen et al. [31]. Briefly, the agar-intralipid solution was made of 1.5% w/v agar powder (Fisher Scientific, UK) and 1% v/v intralipid (20% emulsion, Sigma, UK) in deionised and distilled water. Another dispersion-TM phantom (Fig. 3(2)) with the same geometry was used for ultrasound contrast measurements before and after laser illumination. It was formulated by dispersing the Cy-droplet emulsion at 0.25% v/v in agar-intralipid gel at 36 °C before gelation. The immobility of Cy-droplets in the dispersion-TM phantom allowed separate ultrasound imaging before and after laser scan, necessary because the MSOT system employed was incapable of ultrasound imaging.

### 2.6 Photoacoustic and ultrasound imaging experiment setup

For the photoacoustic imaging with optical vaporisation, the TM agar phantoms were scanned with the MSOT system (inVision 256-TF, iThera Medical). Cy-droplets and the six controls described previously were diluted to 10% relative to stock solution in the distilled/deionised water so as to keep the concentrations of Cyanine7.5 dye identical. The temperature of the water bath was held constant at 34 °C throughout all optical vaporisation experiments. The diluted Cy-droplet solution was introduced into the tubing-TM phantom, and the cross-sections were scanned at three positions (20 mm apart) along the longitudinal direction (Fig. 3 (1)). At each position, photoacoustic imaging was performed at a single wavelength 788 nm (peak absorption of Cyanine7.5) with a fluence of 22.6 mJ/cm^2^ using one pulse (10 ns pulses, 10 Hz pulse repetition frequency) per image, resulting in 30 s acquisition time. For accurate ultrasound contrast assessment before and after laser illumination, the problem of imaging a potentially mobile Cy-droplet solution had to be overcome. To achive this, the dispersion-TM phantom was imaged twice (Fig. 3 (2)). To activate Cy-droplets laden in the phantom, half of the dispersion-TM phantom was scanned longitudinally (along the phantom’s cylindrical axis) with 5 s pulsed laser illumination at each position, with approximately 5 cm total scanning distance and 2 mm step size. The other half of the phantom was used as control. Cross-sectional ultrasound imaging of both halves of the same phantom was performed with a Verasonics V1 system (Verasonics, USA) equipped with an L7-4 (ATL, USA) probe.

For ultrasound imaging with acoustic vaporisation, focused pulses (8 MHz, 10-cycles, 3.39 MPa, mechanical index (MI) = 1.2, pulse-repetition-frequency = 14.3 kHz, total duration of exposure is 8.8 ms) transmitted from a clinical linear array probe L12-5 (ATL, USA) were applied to activate the Cy-droplets. The ultrasound contrast enhancement was quantified using a custom designed ‘imaging-activation-imaging’ sequence [22], [32] on a Verasonics Vantage 256 research platform. Single cycle, low amplitude ultrasound at 4.5 MHz (plane-waves of 15-angle spatial compounding, 106.1 kPa, MI = 0.05) was used at each imaging step to estimate the ultrasound signal level from the contrast agent before and after Cy-droplet activation. The same amount of stock Cy-droplet and blank-droplet emulsion was introduced into a 2L water tank (Fig. 3 (3)) filled with water and equilibrated to 37 °C [33] to achieve a final concentration of approximately 10^6^ droplets/mL. Before each acquisition, the water was mixed to achieve a relatively uniform distribution of droplets. Acoustic absorbers were used to line the water tank to reduce ultrasound reflections.

### 2.7 Photoacoustic image beamforming

The photoacoustic images were presented after beamforming the raw radiofrequency (RF) data extracted from the MSOT ultrasound transducer using a customised Matlab (Mathworks, USA) program. An image was formed by applying a temporal delay for each channel according to different positions along the 270° concave ultrasound transducer array followed by summing each image component over all the 256 channels. The geometry parameters were applied according to the MSOT ultrasound transducer design described by Dima et al. [34].

### 2.8 Data analysis

For the photoacoustic experiment, both the raw RF data and the beamformed photoacoustic images were used to measure the relative photoacoustic signal levels. The first-pulse response of the photoacoustic signal was presented along with six controls. In the case of the beamformed images, the maximum pixel magnitude in the region of interest (ROI) was used as a measure of the relative photoacoustic image signal generated by Cy-droplet vaporisation.

For ultrasound echo signal evaluation, the mean image pixel magnitude in the selected ROI was used to compare the echo signal level before and after the activation of Cy-droplets. For ultrasound imaging with acoustic activation, an ROI was chosen within the Cy-droplet activation area (the focal zone) and used for analysing the signal both before and after activation. The difference in mean pixel magnitude within the ROI before and after the activation was calculated.

For statistical analysis, each experimental result was produced by at least three acquisitions. Student's *t*-test was used to compare the statistical difference between groups with p > 0.05 considered to be not significantly different.

## 3 Results

### 3.1 Cy-droplet and precursor Cy-microbubble characterisation

**Fig. 4:**
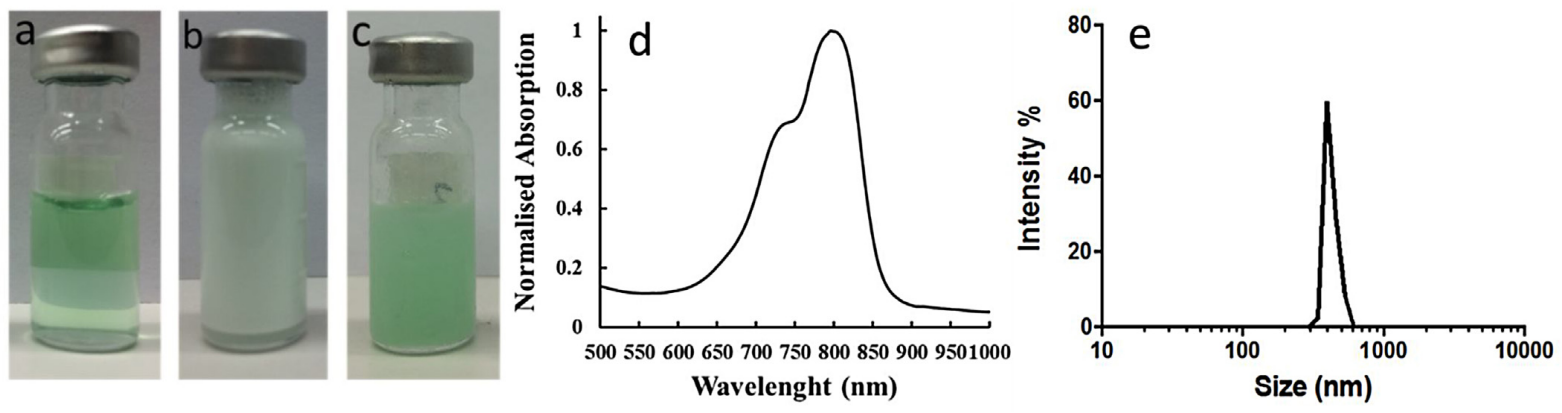
Cy-droplet emulsion preparation and characterisation. (a) 1 mL of Cy-droplet lipid solution with DFB gas sealed in a 2 mL glass vial, (b) precursor Cy-microbubbles after mechanical agitation from Cy-droplet lipid solution, (c) Cy-droplet emulsion after Cy-microbubble condensation, (d) absorption spectrum of the Cy-droplet suspension showing a peak absorption at 788 nm, and (e) size distribution obtained by DLS of the Cy-droplets revealing an average diameter of approximately 400 nm.

**Fig. 5:**
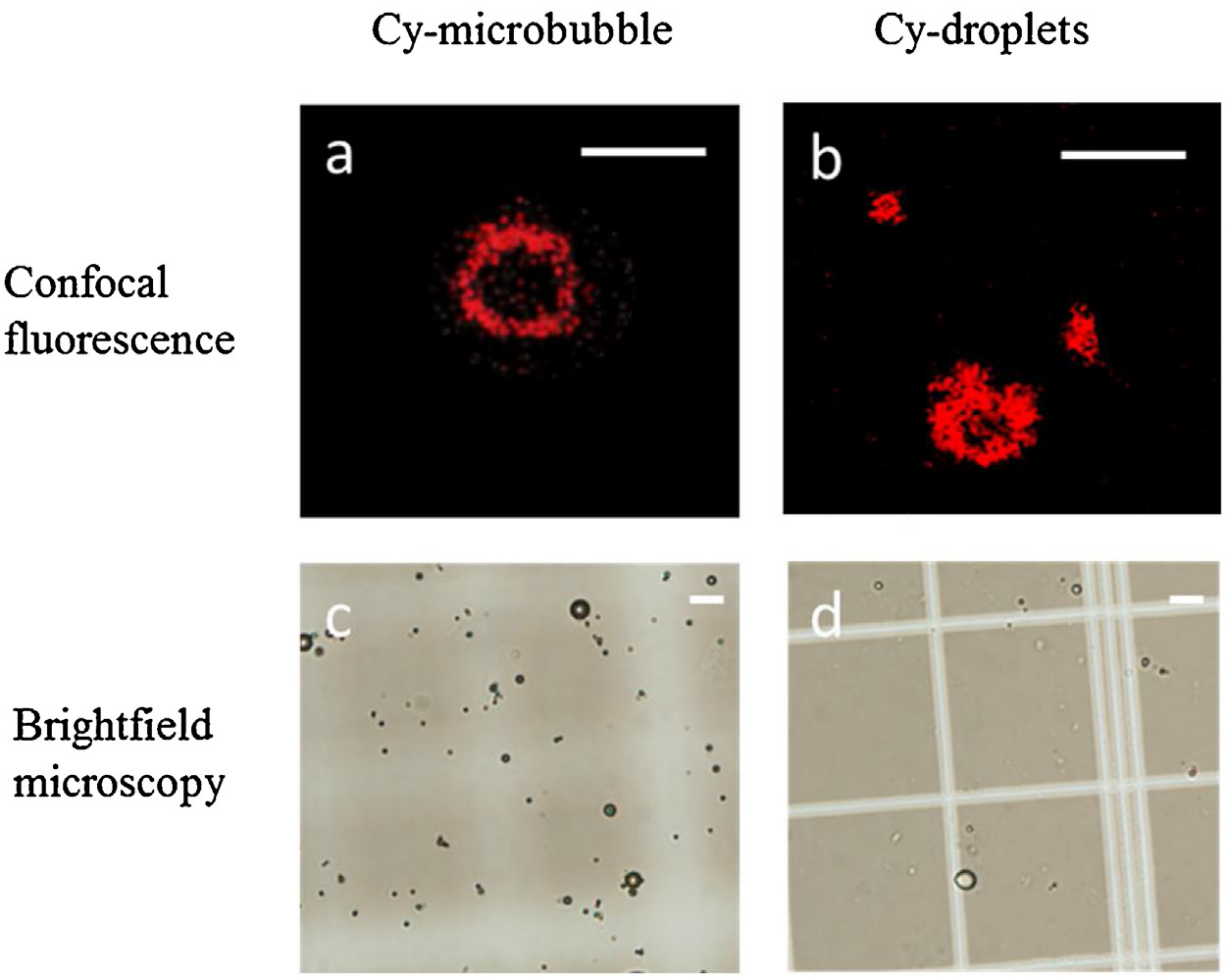
Microscopy of outlier precursor Cy-microbubbles and outlier Cy-droplets (those large enough to be resolvable), presented to illustrate the location of fluorescent lipid. (a, b) Confocal fluorescence of Cy-microbubble and Cy-droplets. (c, d) Bright-field microscopy of Cy-microbubble and Cy-droplets. The scale bars are 10 μm.

The stock precursor Cy-microbubble solution (Fig. 4b) yielded a concentration of ∼5 × 10^9^ microbubbles/mL, and a mean bubble diameter of 1.02 ± 0.40 μm. DLS (Fig. 4e) revealed an average hydrodynamic diameter of approximately 400 nm for both Cyanine-droplets (Fig. 4c) and normal-droplets, with relatively narrow size distributions (polydispersity index = 0.986 and 0.509, respectively). The Cy-droplets are metastable in the liquid state under physiological conditions due to the energy barrier for homogeneous nucleation [35], with a vaporization temperature of 75 °C [36], even though they are superheated. Fig. 5(a and b) show representative confocal microscopic images used to verify the location of fluorescent lipid on precursor Cy-microbubbles and Cy-droplets. The majority of Cy-droplets were beyond resolution limits. The Cyanine7.5 lipid on microbubbles appeared as a circular rim while the Cy-droplets that could be seen demonstrated fluorescence throughout their projected area. The uneven circular projected appearance of Cy-droplets was possibly due to the lipid monolayer of the Cy-microbubble being ‘folded’ or ‘buckled’ after condensation [37]. Fig. 5(c and d) show bright-field micrographs of Cy-microbubbles and large Cy-droplets respectively (again, most Cy-droplets were beyond the resolution limits). The absorption spectrum of the Cy-droplet lipid solution had a peak at around 788 nm (half-maximum waveband ∼710–840 nm) (Fig. 4d).

### 3.2 Photoacoustic signals and imaging contrast of Cy-droplets and controls

**Fig. 6:**
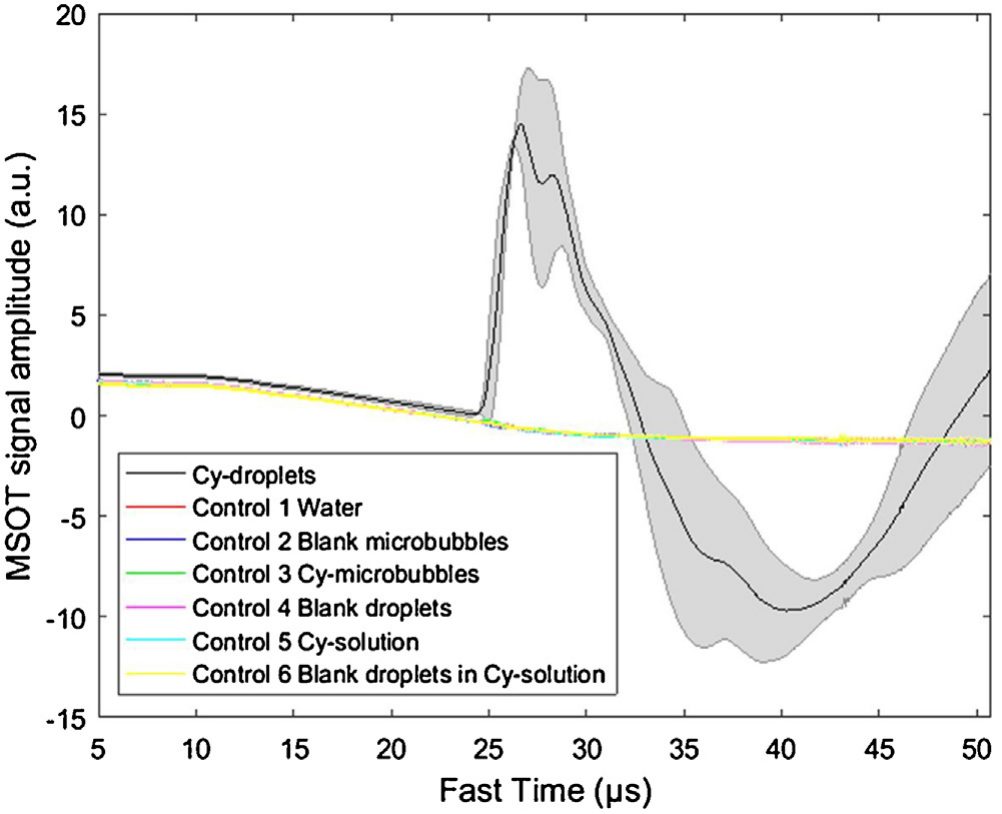
Amplitude of the raw (channel level) photoacoustic signal averaged over 256 elements of the ultrasound ring array, for the first laser pulse illuminating a 10% diluted Cy-droplet solution and six controls. The photoacoustic signal generated from the optical vaporisation of Cy-droplets (black line) produced more than an order of magnitude (maximum mean amplitude 14.5 a.u.) higher signal amplitude than six control groups. The black line and shaded error bar show the mean, and plus and minus one standard deviation, respectively, over three measurements.

The raw photoacoustic signal was plotted as a function of fast time, which was the one-way time-of-flight calculated by using sampling point number of each channel (i.e. 2030 points) and data acquisition sampling frequency (40 MHz) [38]. Fig. 6 plots the mean of the raw photoacoustic signal from 256 channels for the first-pulse response of the Cy-droplets and six controls, with the shaded error bar from three repeats (acquired at three positions showed in Fig. 3(1)). The signal started to rise at about 25 μs corresponding to the position of the tubing-TM phantom. The Cy-droplets produced more than an order of magnitude (maximum mean amplitude 14.5 a.u.) higher signal amplitude than the noise level, whereas none of the six controls produced a detectable photoacoustic signal.

**Fig. 7:**
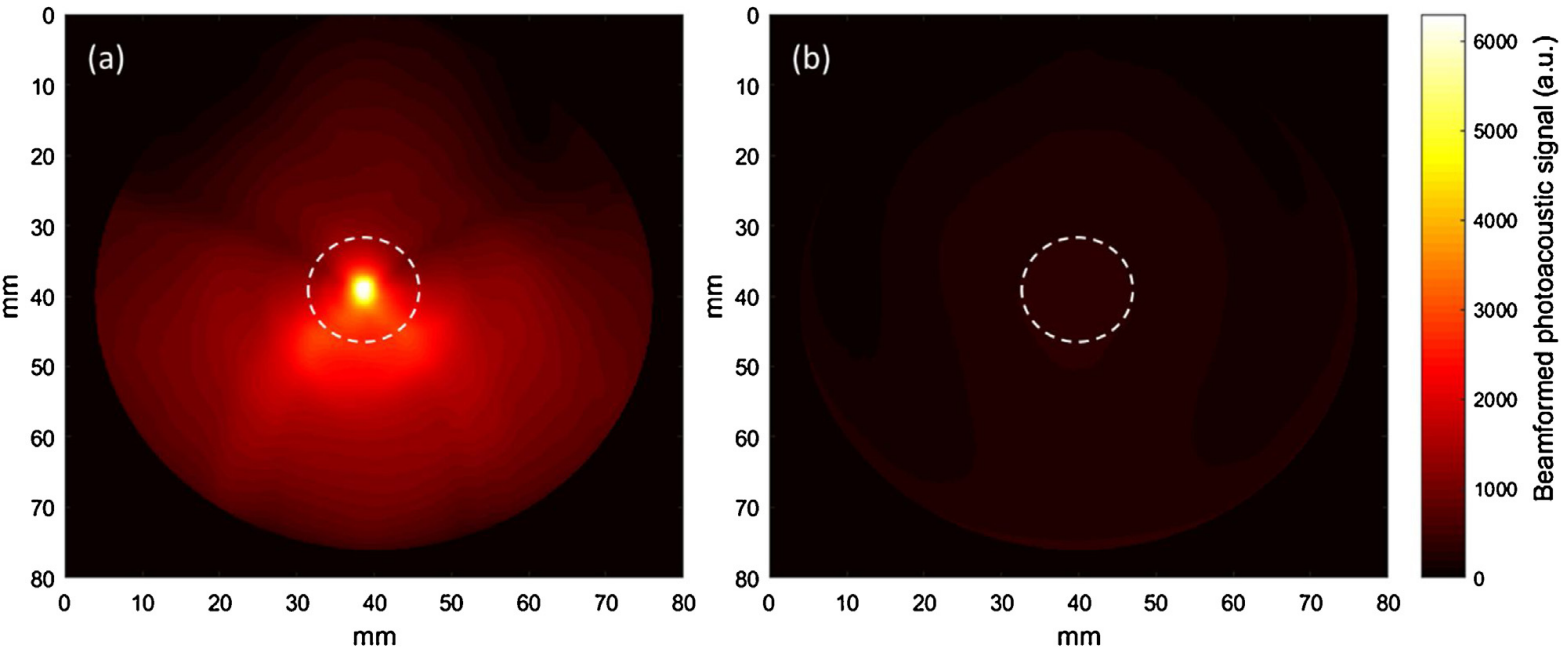
Beamformed photoacoustic images of (a) the first-pulse response of 10% diluted Cy-droplets and (b) that of a representative image of all six controls. The ‘white dashed circle’ shows the ROI for data analysis, which outlines the external circumference of the tubing-TM phantom.

**Fig. 8:**
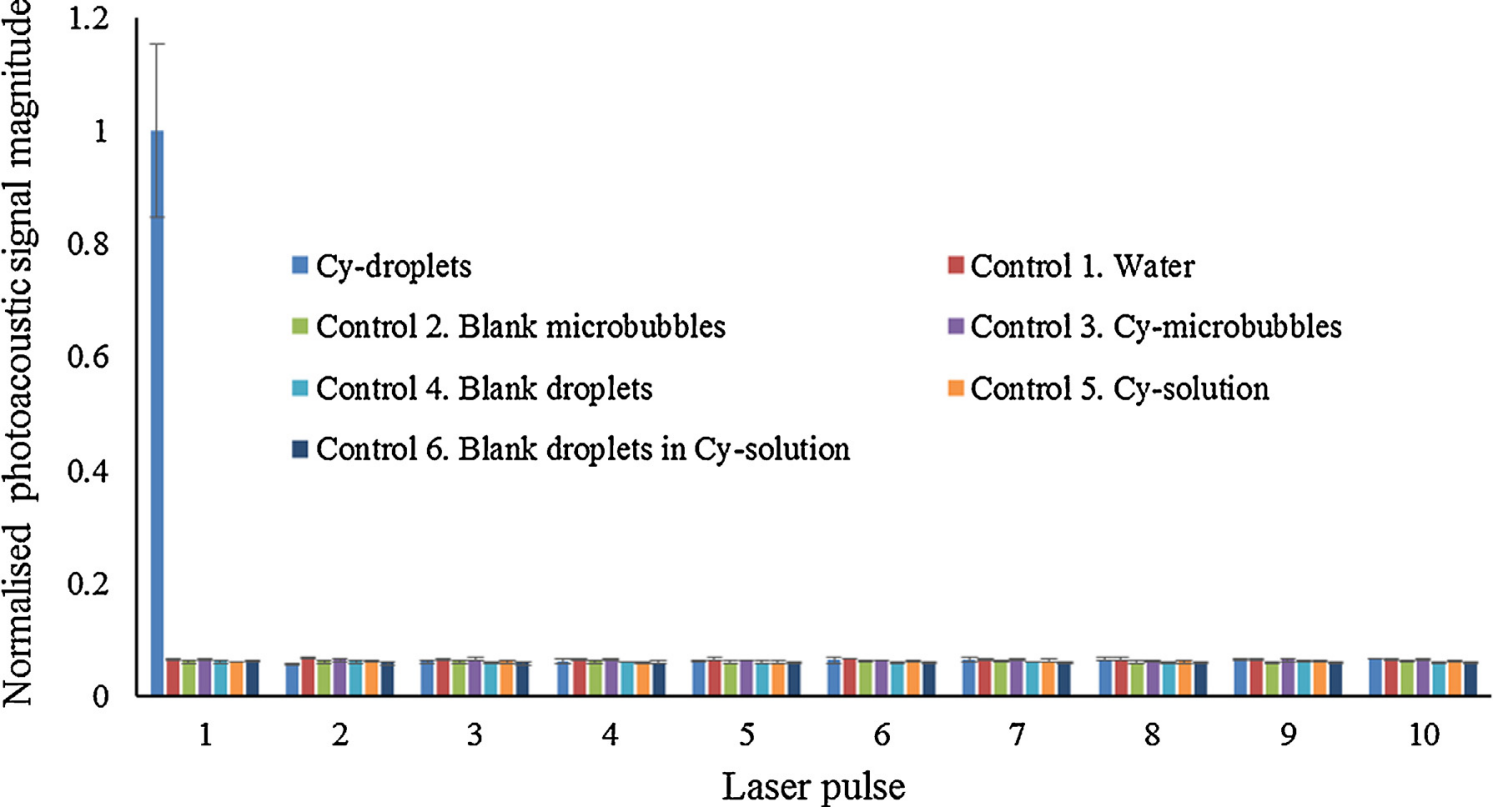
Normalised beamformed photoacoustic image signal amplitude within the analysis ROI for 10% diluted Cy-droplet solution and six controls, showing results for the first ten laser pulses. The first laser pulse vaporised most of the Cy-droplets in the tube, generating substantial photoacoustic signal, while the following pulses, in contrast, produced little signal because few Cy-droplets remained. None of the six controls produced a detectable photoacoustic signal.

**Fig. 9:**
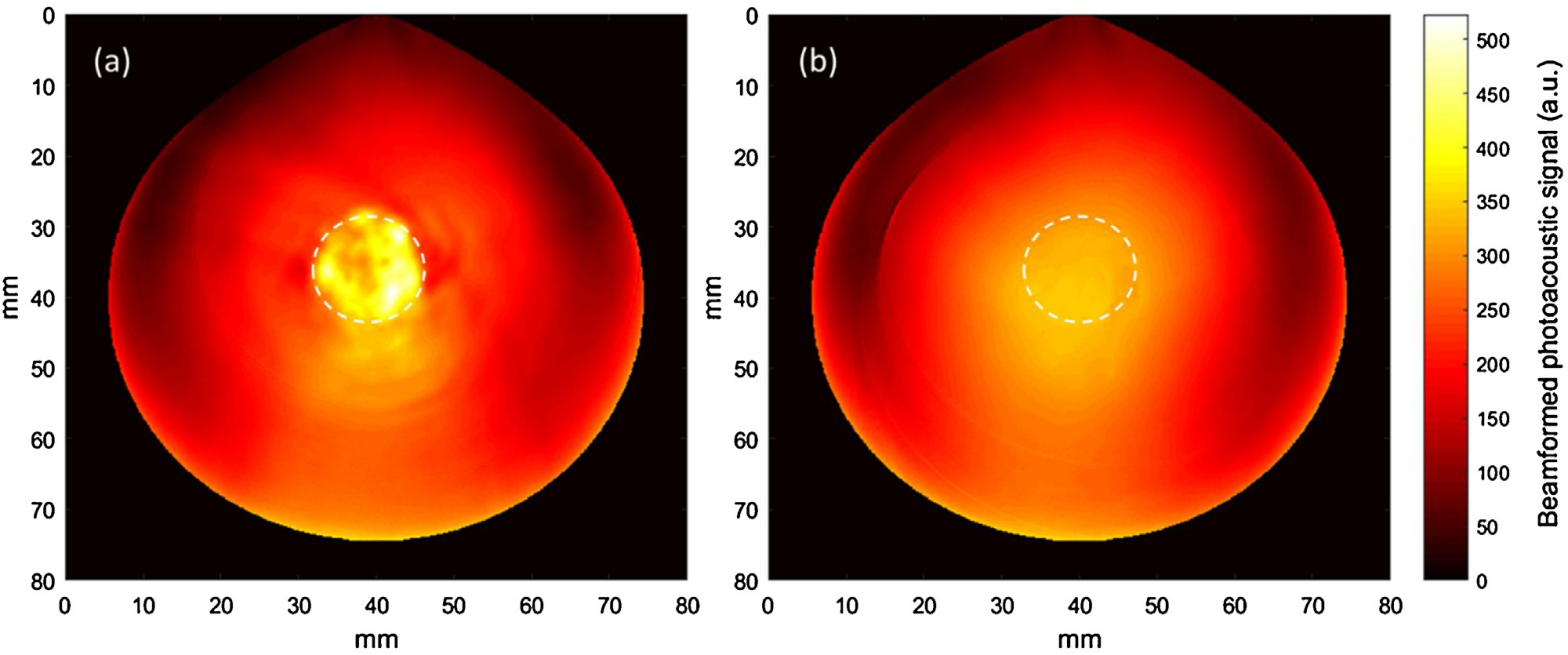
Photoacoustic images of the dispersion-TM phantom showing (a) Cy-droplets (0.25% v/v) and (b) ‘blank’ dispersion-TM phantom control. The optical vaporisation of Cy-droplets immobilised in the phantom induced 8.1 dB higher signal enhancement than the control. The white dashed circles indicate the position of the dispersion-TM phantom and ROI for data analysis.

Typical beamformed photoacoustic images of Cy-droplets and a control are demonstrated in Fig. 7. A 10% diluted Cy-droplet suspension resulted in a 56.3 dB higher enhancement of the spatial maximum imaging signal than six controls. Fig. 8 presents the beamformed photoacoustic imaging signal for the first ten laser pulses. The very first laser pulse vaporised most of the Cy-droplets in the tube, generating substantial photoacoustic signal, while all subsequent pulses produced little signal, possibly because few Cy-droplets were left and suggesting that the enhanced signal was produced by the vaporisation process. Fig. 9 demonstrates that the photoacoustic signal induced by the vaporisation of Cy-droplets (0.25% v/v) immobilised in the dispersion-TM phantom produced 8.1 dB higher signal magnitude than the control (a ‘blank’ TM phantom).

### 3.3 Photoacoustic signal and Cy-droplet concentration

**Fig. 10:**
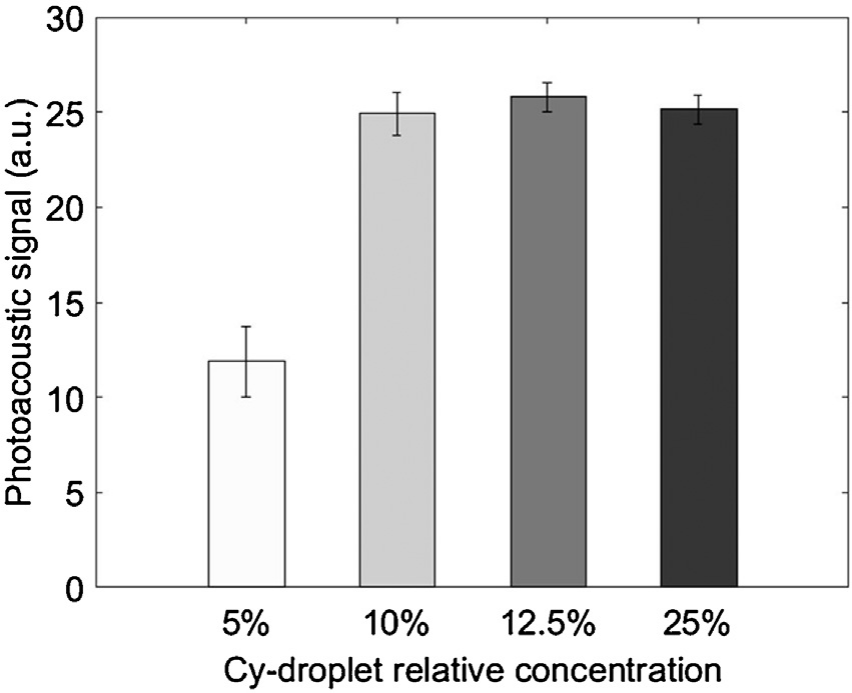
Means of the temporal maximum values across all transducer elements of the magnitude of the first-pulse RF photoacoustic signal, for four different Cy-droplet relative concentrations in the tubing-TM phantom. Clear saturation of the photoacoustic signal occurs above a relative concentration of 10%.

For eventual *in vivo* use, an understanding is needed of the relationship between the concentration of Cy-droplets and the generated photoacoustic signal. Referring to Fig. 10, varying the relative concentration of Cy-droplet solution produced a substantial and significant increase in the first-pulse (vaporisation) photoacoustic signal between 5% and 10% (relative to the stock Cy-droplet solution) but no significant change from 10% to 25%.

### 3.4 Ultrasound echo enhancement via optical vaporisation

**Fig. 11:**
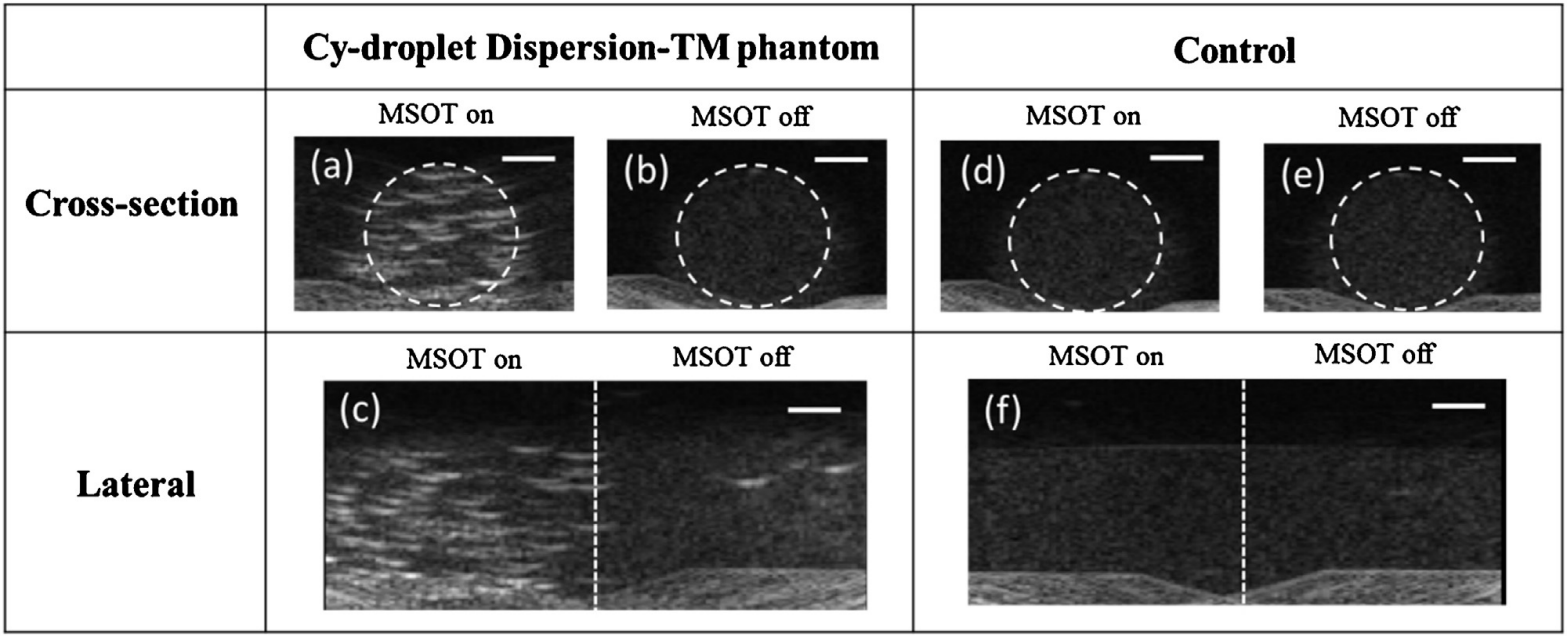
Ultrasound echo enhancement produced by optical activation of Cy-droplets immobilised in the dispersion-TM phantom, and comparison with the control phantom (no droplets). (a, d) cross-sectional echo images of the Cy-droplet laden phantom and control phantom after pulsed laser illumination. (b, e) Cross-sectional images of the Cy-droplet phantom and control where there had been no laser irradiation. (c, f) Longitudinal images of the Cy-droplet phantom and control, in which the white dashed vertical line indicates the boundary between the regions exposed and not exposed to the laser. The band of echoes at the bottom of the images was due to the reflection from an anti-reverberation pad. White dashed overlaid circles highlight the phantom cross-sectional area and the ROI for data analysis. Scale bar is 5 mm.

From the dispersion-TM phantom cross-sectional imaging, the ultrasound echo signal was 11 dB higher where the Cy-droplets had been exposed to pulsed laser illumination (Fig. 11a) than where they had not (Fig. 11b). In Fig. 11c, the longitudinal view of the Cy-droplet laden phantom demonstrates a distinct transition in echo strength at the boundary, indicated by the white dashed overlaid vertical line, between the region that had been (Fig. 11c.left) and that which had not been (Fig. 11c.right) exposed to the laser. An equivalent phantom with no droplets or particles of any kind embedded (Fig. 11d, e, f) provided confirmation of the lack of echo signal from the background material of the phantom, and that this did not change with exposure to the laser. The echo signal layer at the bottom of images was due to the reflection from an acoustically absorbing pad on which the phantom was placed, used to reduce acoustic reverberations.

### 3.5 Ultrasound imaging with acoustic vaporisation of droplets

**Fig. 12:**
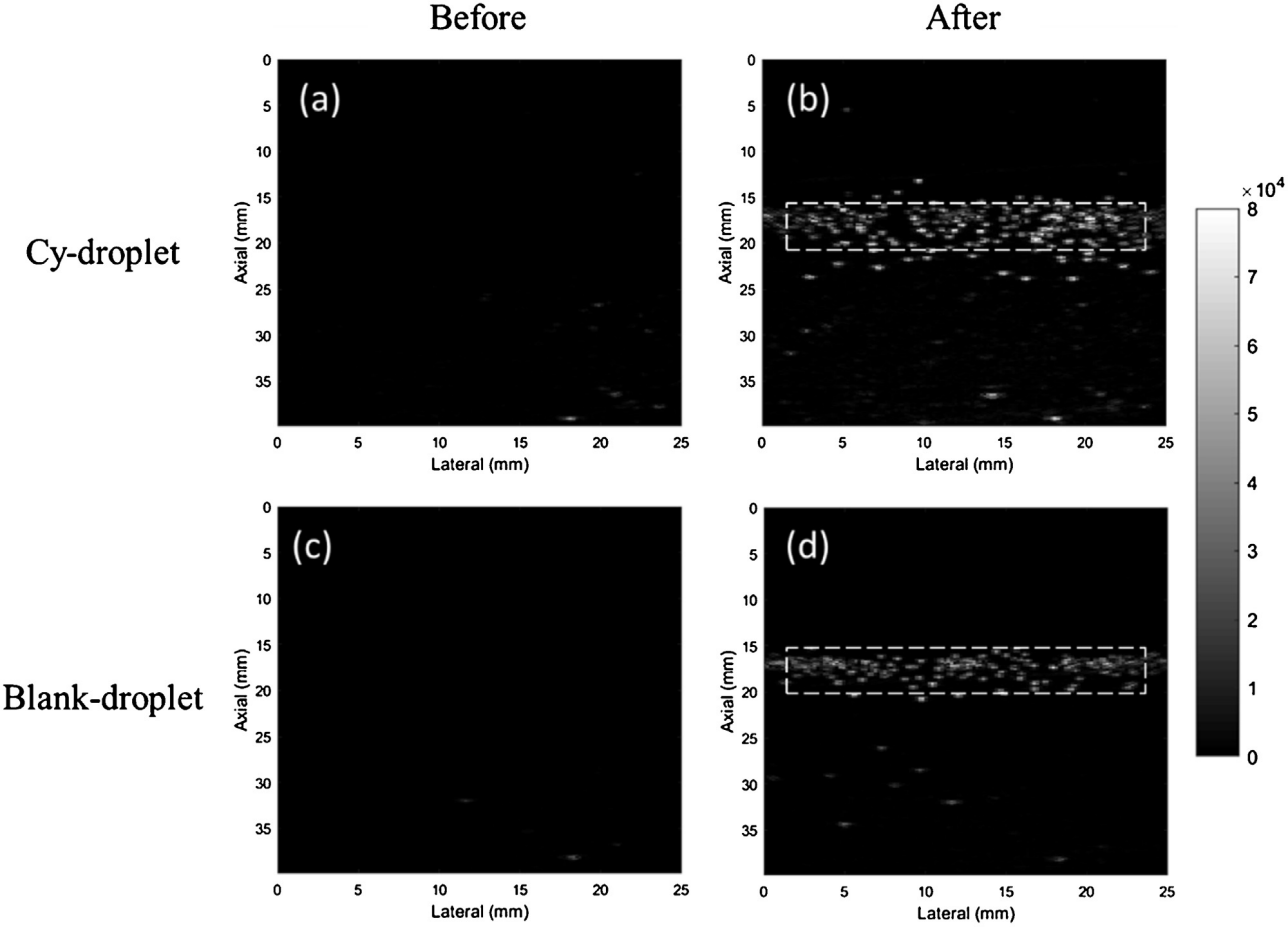
Ultrasound echo enhancement with acoustic vaporisation. (a, b) Cy-droplets and (c, d) blank-droplets (no cyanine dye in the lipid shell). Representative ultrasound images are shown before (a, c) and after (b, d) acoustic vaporisation in a 2L 37 °C water tank. The dashed white rectangular area was the ROI applied to quantify the echo strength.

**Fig. 13:**
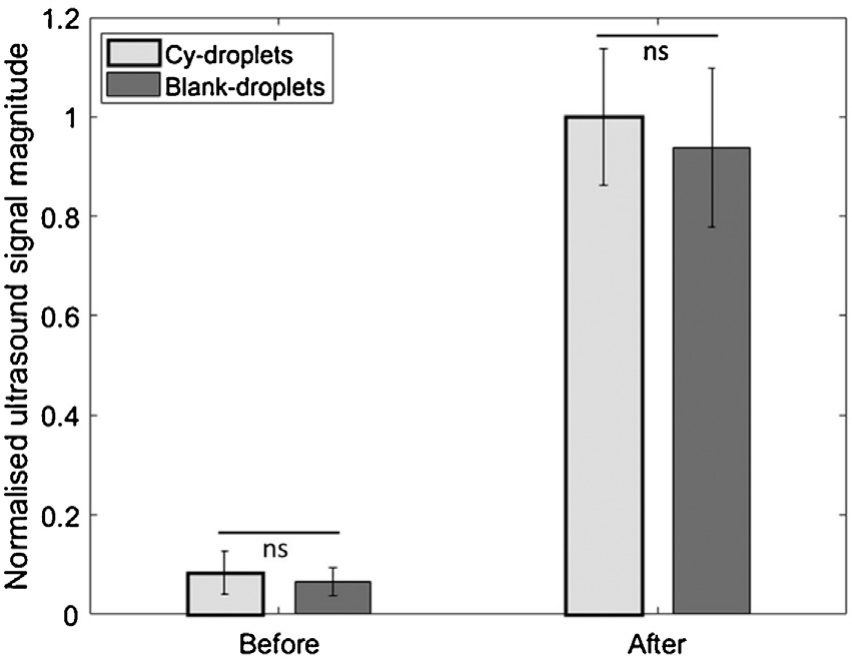
Quantitative comparison of relative ultrasound echo strength before and after acoustic vaporisation of Cy-droplets and blank-droplets (no dye) in water at 37 °C. There was no significant difference (‘ns’) between the echo strength from Cy-droplets and blank-droplets, either before (p = 0.46) and after (p = 0.56) acoustic vaporisation. Vaporisation caused more than a ten-fold increase in echo strength.

Fig. 12 shows representative ultrasound images before and after acoustic vaporisation of droplets. Increased echo signal after acoustic vaporisation appeared around the pre-set focusing depth (16 mm) of the vaporising pulses and generated an average of 11.98 and 14.39-fold echo amplitude enhancement for Cy-droplets (Fig. 12a, b) and blank-droplet controls (Fig. 12c, d) respectively, where the quantitative comparison is provided by Fig. 13. There was no significant difference in the results between Cy-droplets and control blank-droplets (p > 0.05). A few echoes were seen before acoustic vaporisation, usually deep in the water tank (Fig. 12a, c), and outside the focal depth after vaporisation, most frequently immediately below the focal zone (Fig. 12b, d).

Data underlying this article is available on request: please contact ultrasound-imaging-group@imperial.ac.uk. A general licence applies to all users of the data.

## 4 Discussion

### 4.1 Overall results

When viewed in totality, the results demonstrate that the Cy-droplets represent a new dual-triggerable and dual-modality sub-micron phase-change contrast agent, which can be activated by a pulsed laser or by diagnostic ultrasound pulses to offer both photoacoustic and ultrasound signal enhancement via optical vaporisation or by ultrasound echo imaging of the resultant gaseous bubbles. Further work is needed to investigate aspects of the results in more detail and to determine optical, acoustic and signal processing parameters for optimised use.

### 4.2 Comparison with other optically activated phase change and dual-mode agents

Other optically activated phase change agents that produce dual-mode (photoacoustic and ultrasound) imaging include those of Hannah et al. [11], who incorporated indocyanine green (ICG) into the albumin shells of nanodroplets, and Wei et al. [19], who used a nanoemulsion in which shell-less droplets were coated with gold nanospheres. The advantages and disadvantages of each approach have yet to be fully uncovered and studied. ICG seems to be required in ∼mM amounts (2 mM ICG was used in [11]) but has the advantage that it is already approved for clinical use, and the short lifetime (a few μs [19]) of the cavitation bubbles generated by pulsed illumination of the nanoemulsion may not be ideal for ultrasound imaging. Ours is the first investigation of whether a combined photoacoustic and ultrasound contrast agent can be generated by condensing precursor fluorescent microbubbles containing a low b.p. perfluorocarbon (i.e. DFB). In principle, any suitable dye (or nanoparticle) could act as the optical absorber in the precursor microbubble shell. Here we used Cyanine7.5 attached by bioconjugation. In combination with DFB, this provided highly efficient triggering of vaporisation by a commercially available photoacoustic imaging system, with only ∼nM amounts of the dye (13 nM in this work). The system is also versatile; our use of commercially available DSPE-PEG(2000)-NH_2_ for bioconjugation enables easy attachment of targeting ligands (e.g. folate for intracellular delivery [39]) and/or complementary imaging units (e.g. Gadolinium(III)-DOTA) to incorporate additional functionalities. Finally, as noted in [39], the use of DFB also enables efficient use in the third imaging mode, acoustic triggering of vaporisation, at an FDA approved MI consistent with clinical use. All other published microbubble or droplet based dual contrast agent studies that we could find have very different objectives and modes of action, involving fluorescent dyes to provide either dual ultrasound-fluorescence imaging (e.g. [40], [41]) or dual fluorescence and magnetic resonance imaging (e.g. [42]), making direct comparison with the present work inappropriate.

### 4.3 Concentration dependence

Changing the Cy-droplet relative concentration from 5% to 10% nearly doubled the photoacoustic signal enhancement (Fig. 10), probably because a greater number of Cy-droplets yielded more optical vaporisation events [22] producing a higher density of acoustic sources. Further study is required to determine the linearity and concentration range of this dependence. Saturation of the photoacoustic signal enhancement above a relative concentration of 10% is not at present fully understood, and requires further study, but may be associated with signal saturation within the photoacoustic detection system.

### 4.4 Optical activation threshold energy

It is helpful to know that, as demonstrated here, standard laser pulses used for photoacoustic imaging were sufficient to activate the Cy-droplets, and that this was achieved with a laser fluence that was below the safe limit (100 mJ/cm^2^) for human skin exposure [12]. Nevertheless, further lowering of the threshold for vaporisation, while preserving general stability, is preferable to enable deep tissue optical activation. More detailed studies are needed of the dependence of the optical vaporisation threshold on laser exposure parameters such as fluence, and on droplet properties. For example, the larger the droplet the lower the laser fluence needed to achieve phase transition [12]. In this study, a polydispersed precursor microbubble size distribution produced polydispersed droplets. In future studies it would be desirable to select the size of droplets [28], to investigate the optical activation threshold energy as a function of size. It is also important to optimise the amount of Cyanine7.5 on the lipid membrane of precursor microbubbles, since this will affect the optical activation threshold. Further tuning may be achieved by altering the boiling point of the gas, or combinations of gases, in the precursor bubbles [43]. Finally, the lipid composition of the shell, particularly lipid acyl chain length, can affect phase-change activation energy [36], suggesting opportunities, for example, to replace 1,2-dipalmitoyl-sn-*glycero*-3-phosphocholine (DPPC, C16) with a lipid possessing a shorter acyl chain, e.g., 1,2-dimyristoyl-sn-*glycero*-3-phosphocholine (DMPC, C14).

### 4.5 Selection of optical absorber and possibilities for “droplet recognition imaging”

The peak absorption wavelength of Cyanine7.5 (788 nm) is within the ‘imaging optical window’ of biological tissue (600–1300 nm) [44] with minimal tissue attenuation that enables deep penetration of light, making the Cy-droplets amenable to enhancing the sensitivity of whole body small animal vascular and molecularly targeted imaging. Although ICG also has absorption in this window, with the additional advantage that it is approved for clinical use, it has poor photostability, a molar absorption coefficient [29] that is much lower than that of Cyanine7.5 (over 200,000 mol^−1^cm^−1^L) and its fluorescence emission is of no value in the present context which uses only the absorbed energy (the relatively low quantum yield of Cyanine7.5 contributes to its efficient transfer of optical energy to heat for vaporisation). Finally, Cyanine7.5 was shown to have quite a narrow absorption spectrum (710–840 nm). This may provide opportunities for future work to explore whether the optical vaporisation threshold can be adjusted so that only a narrow range of wavelengths will induce a phase change and thus elicit a photoacoustic or ultrasound signal enhancement. If this were shown to be the case, it brings about two important possibilities. First, changing the optical wavelength may allow a limited form of vaporisation spectroscopy for photoacoustic imaging of the agent (e.g. starting at wavelengths not expected to cause a phase transition), so that the method may go beyond simple signal enhancement to agent recognition imaging, by analogy to the way that nonlinear (e.g. pulse-inversion) techniques have taken conventional microbubble ultrasound imaging beyond simple blood echo signal enhancement. Admittedly, such tissue background-suppressed imaging of the agent might be achieved (in the absence of significant tissue motion) also by vaporisation contrast subtraction imaging, but there may be advantages in combining both temporal and wavelength subtraction approaches. Second, the whole cyanine dye family (e.g. from Cyanine3 to Cyanine7.5) is commercially available, offering a range of peak absorption wavelengths and raising the tantalising possibility of co-administering various Cy-droplet types into the blood stream, each with different activation wavelengths for differential droplet recognition. This may be useful if, for example, each droplet type were functionalised to bind to a different molecular target. These features make cyanines very promising candidates for the optical absorber, particularly during this research phase of the work. Although cyanines have not been approved by FDA for clinical use, other dyes, or nanoparticles, could be easily substituted and may offer similar possibilities.

### 4.6 Synergistic effect of combining ultrasound and optical energy for lowering the vaporisation threshold

Previous studies have shown that the simultaneous deposition of optical (laser illumination) and acoustic (ultrasound rarefactional pressure) energy can lower exposure thresholds to achieve enhanced photoacoustic and/or acoustic signal from various contrast agents [45], [46], [47]. Future work will involve investigating the vaporisation thresholds of Cy-droplets excited with a nanosecond laser pulse coinciding with various phases of an ultrasound activation wave. In particular, based on the observation in [48], that vaporisation microbubbles can emerge from droplets through the first rarefactional phase of an ultrasound pulse, we hypothesise that the vaporisation threshold can be most reduced by aligning laser pulses to a rarefactional phase. Such synergism could be harnessed to improve vaporisation imaging depth, as well as sensitivity and specificity of targeted molecular imaging and therapy using Cy-droplets.

### 4.7 Acoustic vaporisation thresholds

Echoes seen before acoustic vaporisation (Fig. 12a, c), suggest spontaneous vaporisation of some droplets. These were usually spotted deep in the water tank, and therefore may correspond to larger droplets which would more readily undergo spontaneous phase change. This would be consistent with the echoes that appeared outside the focal depth after vaporisation, which most frequently appeared immediately below the focal zone (Fig. 12b, d), i.e., they were possibly due to acoustic vaporisation of the largest droplets which would have had the lowest acoustic vaporisation thresholds compared with the majority of droplets. Future studies are required to fully characterise the acoustic vaporisation.

## 5 Conclusion

In this study, we have demonstrated the development and characterisation of an optically and acoustically triggerable sub-micron phase-change contrast agent ‘Cy-droplets’ manufactured with a highly volatile perfluorocarbon via the ‘microbubble condensation’ approach. For optical droplet activation, Cy-droplets generated substantial photoacoustic transient signal from the vaporisation light pulse, and gas bubble formation thereafter provides stable enhanced ultrasound signal. For acoustic activation, Cy-droplets can be vaporised using external acoustic energy with clinical diagnostic ultrasound pulse parameters, offering ultrasound echo imaging contrast. This versatility offers photoacoustic-ultrasound dual imaging and high selectivity, which would benefit cancer molecular imaging and targeted drug delivery using Cy-droplets.

## Conflict of interest

The authors declare that there are no conflicts of interest.
