# Supplementary material for: Optically and acoustically triggerable sub-micron phase-change contrast agents for enhanced photoacoustic and ultrasound imaging
Source: Photoacoustics. 2017 Apr 11;6:26–36. doi: 10.1016/j.pacs.2017.04.001 (PMC5423321; doi:10.1016/j.pacs.2017.04.001)
Supplement: Supplementary file 1 [file mmc1.docx]

# Supporting information

The DSPE-PEG(2000)-cyanine7.5 formation analysis:

1H NMR (500 MHz, CDCl3) for DSPE-PEG(2000)-cyanine7.5: δ 0.87 (t, CH3, 6H, J = 0.87 Hz), 1.25 (s, CH2 , 56H), 1.50-1.66 (m, CH2CH2CO + CH2, 6H), 1.72-1.86 (m, CH2, 4H), 1.95-1.99 (m, CH2, 2H), 1.99 (s, CH3, 6H), 2.00 (s, CH3, 6H), 2.30 (m, CH2CH2CO, 4H), 2.51-2.62 (m, CH2, 6H), 3.44 (m, CH2CH2N, 2H), 3.53-3.59 (m, CH2N+, 2H), 3.64 (s, PEG ≈ 140 H), 3.72 (s, CH3N, 3H), 3.96 (m, CH2CH2NH, 2H), 4.22 (m, trans-PO4CH2CH, 1H), 4.32 (m, COOCH2COH, 2H), 4.40 (m, cis-PO4CH2CH, 1H), 5.26 (m, PO4CH2CH ,1H), 5.95-6.07 (t, CH, 1H, J = 6.01 Hz), 6.09-6.19 (d, CH, 1H, J = 6.13 Hz), 6.29 (bs, NHCOOCH2, 1H), 6.96 (bs, NHCOCH2, 1H), 7.32-7.51 (m, CH, 5H), 7.56-7.80 (m, CH, 4H), 7.88-7.99 (m, CH, 4H), 8.05-8.15 (m, CH, 2H). UPC2/MS τr= 4.57 min; m/z for [C179H323N6O56P]3+ expected = 1161.41, found = 1161.84 (ESI+). UV−vis (H2O): λmax (nm) [εmax] (M−1· cm−1): 796 [222,000], 738 sh [31,800].


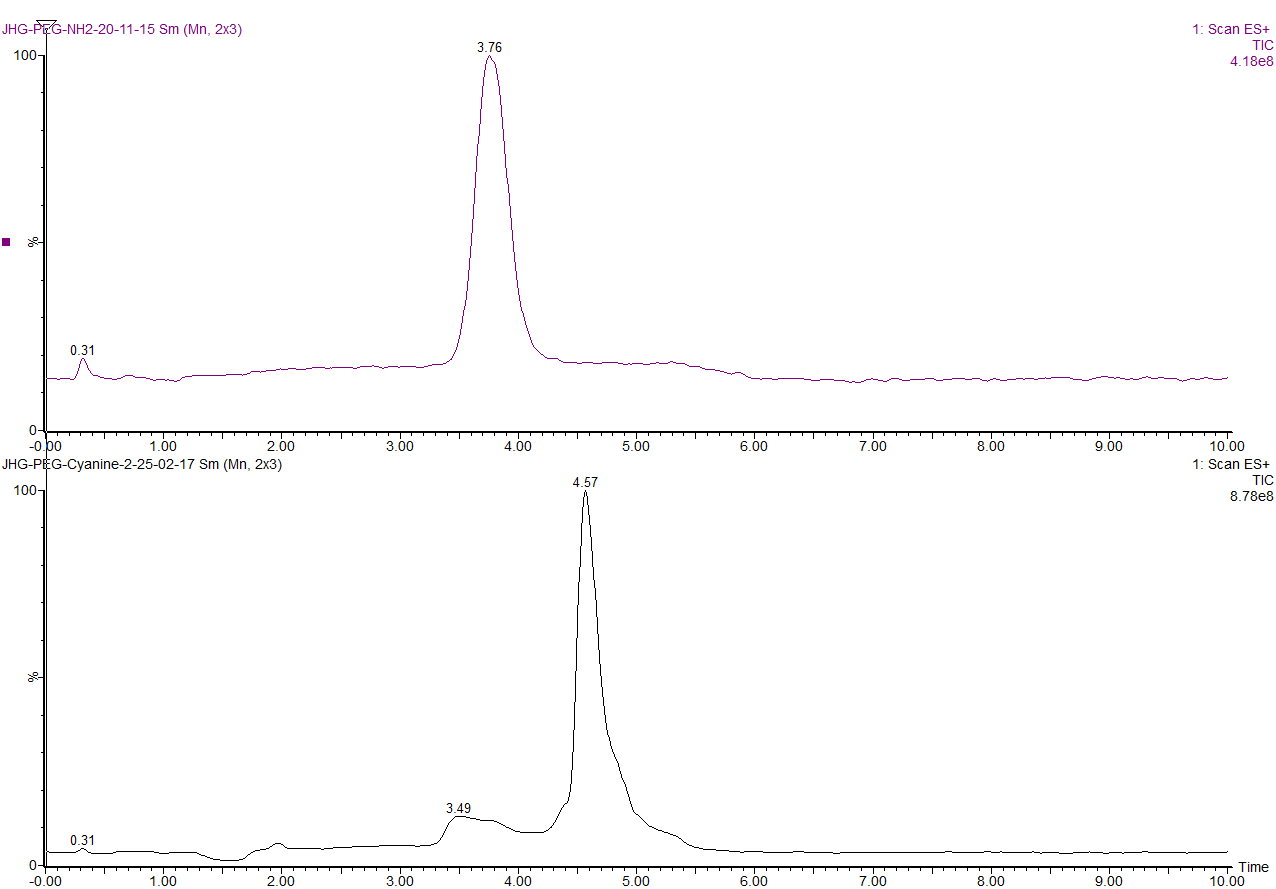


**Figure S1**. Typical total ion chromatograms obtained for DSPE-PEG(2000)-cyanine 7.5 after dialysis purification (*bottom*) and initial DSPE-PEG(2000)-NH_2_ (*top*, control).


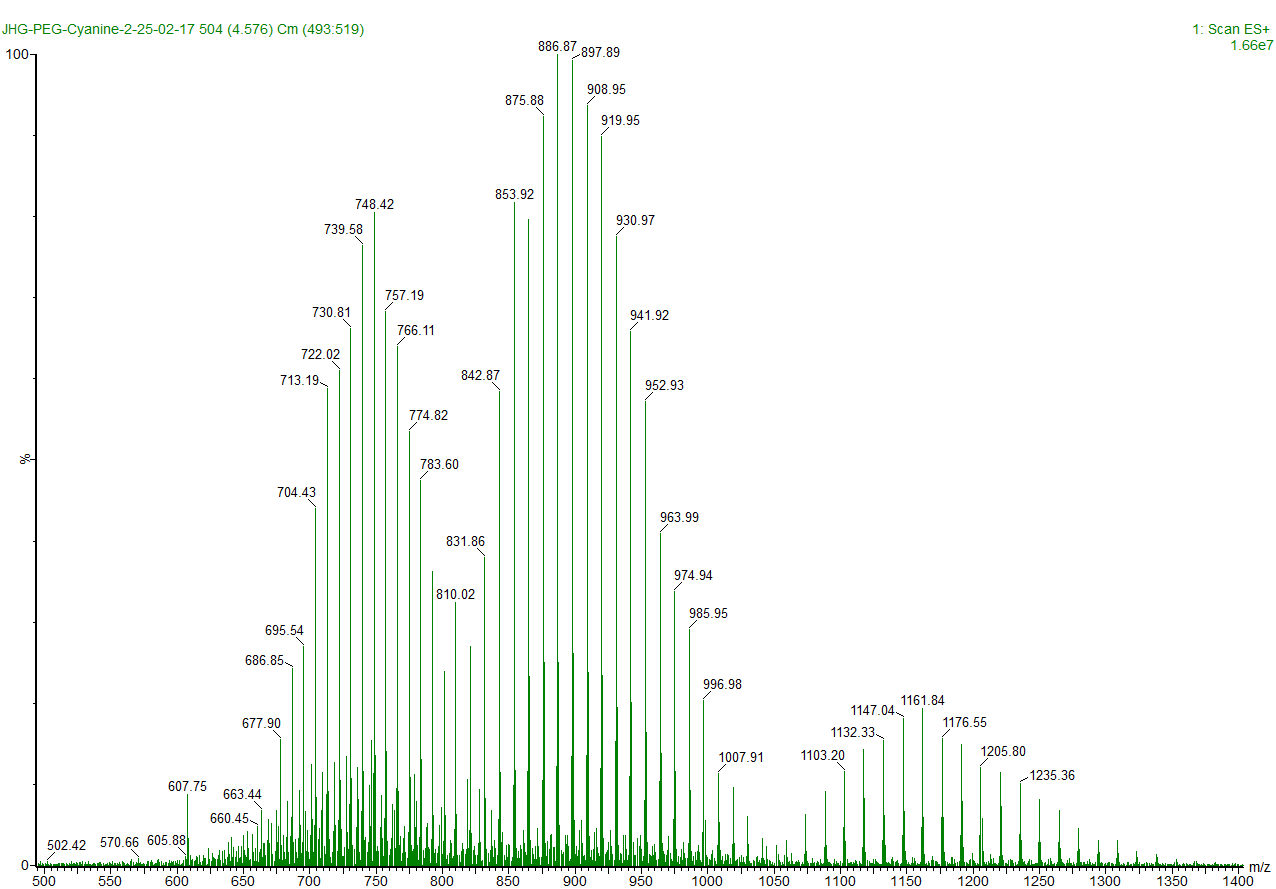


[DSPE-PEG_x_-cyanine 7.5 + 2 H^+^ + 2 NH_4_^+^]^3+^

**Figure S2**. Positive ESI-MS m/z spectra of DSPE-PEG(2000)-cyanine 7.5 product: m/z for [DSPE-PEG(2000)-cyanine 7.5 + 2H^+^ + 2NH_4_^+^]^3+^ (C_179_H_323_N_6_O_56_P) expected = 1161.41, found = 1161.84.
